# Behavioral Analysis of Visitors to a Medical Institution’s Website Using Markov Chain Monte Carlo Methods

**DOI:** 10.2196/jmir.5139

**Published:** 2016-07-25

**Authors:** Teppei Suzuki, Yuji Tani, Katsuhiko Ogasawara

**Affiliations:** ^1^ Faculty of Health Sciences Graduate School of Health sciences Hokkaido University Sapporo Japan; ^2^ Department of Medical Informatics and Hospital Management Asahikawa Medical University Asahikawa, Hokkaido Japan

**Keywords:** information-seeking behavior, Internet, media, social, Bayesian analysis, Web marketing

## Abstract

**Background:**

Consistent with the “attention, interest, desire, memory, action” (AIDMA) model of consumer behavior, patients collect information about available medical institutions using the Internet to select information for their particular needs. Studies of consumer behavior may be found in areas other than medical institution websites. Such research uses Web access logs for visitor search behavior. At this time, research applying the patient searching behavior model to medical institution website visitors is lacking.

**Objective:**

We have developed a hospital website search behavior model using a Bayesian approach to clarify the behavior of medical institution website visitors and determine the probability of their visits, classified by search keyword.

**Methods:**

We used the website data access log of a clinic of internal medicine and gastroenterology in the Sapporo suburbs, collecting data from January 1 through June 31, 2011. The contents of the 6 website pages included the following: home, news, content introduction for medical examinations, mammography screening, holiday person-on-duty information, and other. The search keywords we identified as best expressing website visitor needs were listed as the top 4 headings from the access log: clinic name, clinic name + regional name, clinic name + medical examination, and mammography screening. Using the search keywords as the explaining variable, we built a binomial probit model that allows inspection of the contents of each purpose variable. Using this model, we determined a beta value and generated a posterior distribution. We performed the simulation using Markov Chain Monte Carlo methods with a noninformation prior distribution for this model and determined the visit probability classified by keyword for each category.

**Results:**

In the case of the keyword “clinic name,” the visit probability to the website, repeated visit to the website, and contents page for medical examination was positive. In the case of the keyword “clinic name and regional name,” the probability for a repeated visit to the website and the mammography screening page was negative. In the case of the keyword “clinic name + medical examination,” the visit probability to the website was positive, and the visit probability to the information page was negative. When visitors referred to the keywords “mammography screening,” the visit probability to the mammography screening page was positive (95% highest posterior density interval = 3.38-26.66).

**Conclusions:**

Further analysis for not only the clinic website but also various other medical institution websites is necessary to build a general inspection model for medical institution websites; we want to consider this in future research. Additionally, we hope to use the results obtained in this study as a prior distribution for future work to conduct higher-precision analysis.

## Introduction

To reduce the existing “asymmetry of information” between a patient and physician, patients routinely access Web-based information about their health problems and medical treatment options. Internet-based medical resources are constantly being developed and expanded on such an environment [1]. Consistent with the “attention, interest, desire, memory, action (AIDMA)” model of consumer behavior, patients collect information about the medical institutions available using the Internet to select information for their particular needs [2].

Because of this situation, recently, many medical institutions are intent on improving their websites. With the development in Internet environment and devices, we are now able to obtain information on many medical institutions in diverse ways. Many companies achieve greater advertising effects by active release of information on the Internet. Therefore, medical institutions actively using social media are also increasing. However, such actions and study are insufficiently advanced in Japan.

Market researchers and social psychologists routinely conduct various consumer behavior analyses based on the AIDMA model to predict factors influencing consumer action and purchase decisions and clarify consumer psychology and internal states [3].

Studies of consumer behavior may be found in areas other than medical institution websites. After the expansion of Web advertisements and publicity, Internet sales greatly increased the rate of Internet usage. Research can be conducted using the Web access logs on visitor search behavior. To investigate product sales over the Internet, searching behavior models such as the “search keyword” and “page view” assume that the searches are an expression of consumer needs. In this study, we determined the probability of visits to a certain webpage by the search keyword using the Markov Chain Monte Carlo (MCMC) methods [4,5]. Recently, marketing research has yielded positive results applying Bayesian statistics with improvements in computer count ability and expects to apply them to a greater degree in the future [6-8]. At this time, research applying the patient searching behavior model to medical institution website visitors is lacking.

In this study, we have developed a hospital website search behavior model using a Bayesian approach to clarify the behavior of medical institution website visitors and determine the probability of their visits classified by the search keyword.

## Methods

### Subject

**Figure 1:**
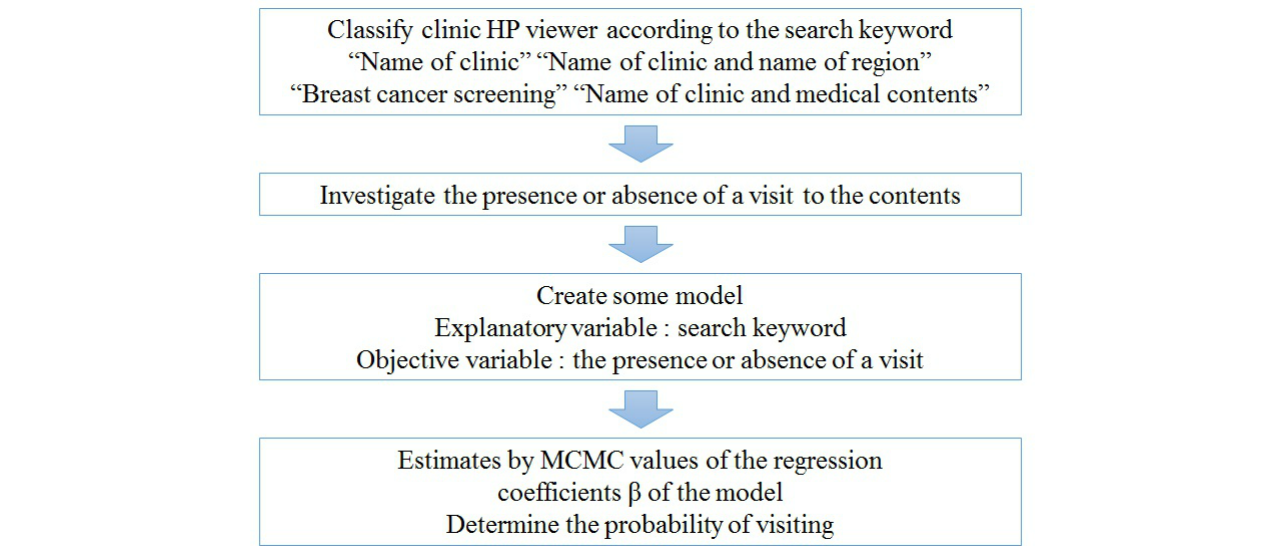
Flowchart.

The flowchart we propose for our research is shown in Figure 1.

We used the website data access log of a clinic of internal medicine and gastroenterology in the Sapporo suburbs for our research, collecting data (336 cases) from January 1 through June 31, 2011. We used Google Analytics to analyze the data access log [9]. The contents of the 6 website pages included the following: home, news, content introduction for medical examinations, mammography screening, holiday person-on-duty information, and other. We used all pages in the clinic for this study. The other page introduces the communication space attached to a hospital. A second visit to the website during the same visit session, distinguished from the first visit and to be counted more correctly as an index page, we classified as “the website (again).” The search keywords we identified as best expressing website visitor needs were listed as the top 4 headings from the access log: clinic name, clinic name + regional name, clinic name + medical examination, and mammography screening.

#### Methods of Analysis

**Figure 2:**
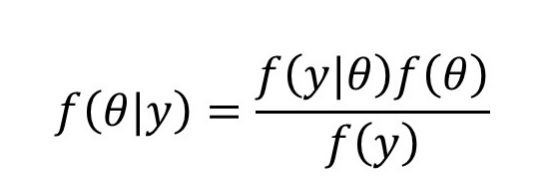
Bayes' theorem.

In this study, we applied Bayes’ theorem as the analysis method. The obtained data were y, and the parameter was defined as θ. Both were random variables and are expressed using Bayes’ theorem as shown in Figure 2.

**Figure 3:**
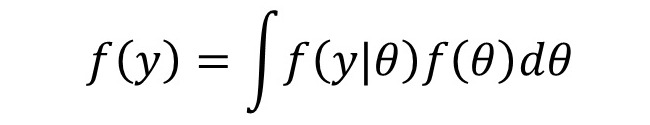
Distribution of the data -f(y).

The left-hand side was called the posterior distribution. This represented the distribution of θ when data y were obtained. The right-hand side of *f* (y|θ) was the likelihood, and *f* (θ) was the distribution of θ. This distribution was called the prior distribution. The distribution of the data expressed by the following equation was represented by *f* (y). We analyzed using this method as shown in Figure 3.

**Figure 4:**
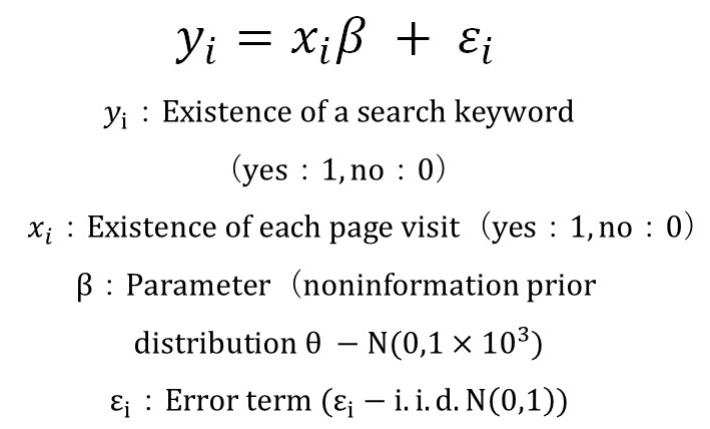
Binomial probit model.

Using the search keyword as the explaining variable, we built a binomial probit model allowing the inspection of the contents of each purpose variable [10]. The binomial probit model is a discrete selection model used in marketing science. In our study, this model used the formulas as described in Figure 4.

The discrete selection model was formulated to address the behaviors of individuals choosing alternatives from their selection sets. In marketing science, this concept is applied to verify consumer selection behavior [11].

Using this model, we determined the beta value and generated a posterior distribution, showing the visit probability to each category classified by the search keywords.

We performed the simulation using MCMC with a noninformation prior distribution for this model and determined the visit probability classified by keyword for each category. We used the Gibbs sampling method, sampling 50,000 times. We also canceled the first 5000 samples, as an initial dependence period (burn-in) [12].

**Figure 5:**
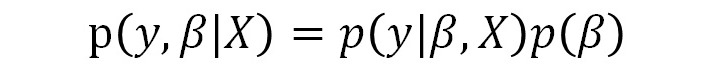
Joint distribution.

The joint distribution is expressed as shown in Figure 5.

Generally, to check the convergence of the sampling, autocorrelation function (ACF) is used. Thus, in this study, we used ACF to check sample convergence. With the vertical axis as the autocorrelation coefficient, when autocorrelation is high, the accuracy of the Markov chain is low.

Although the form of the ACF in the determined posterior distribution and convergence was observed, there was a problem in reproducibility. In this research, the log judged precedence research to reference completed by 30 or more and the auto correlation coefficient or less by 0.1. In this research, we used statistical software R (version 2.13.0) for the simulation analysis [12,13].

#### Definition of Visit Probability

To evaluate the posterior probability density function presumed by MCMC, we used the highest posterior density (HPD) interval. As the value was computed using the Bayesian approach and one of the point estimates, the value alone was not sufficient for evaluation purposes. Therefore, for the interval estimate, because all HPDs of the obtained frequency function were either positive or negative, we assumed this was significant and defined the median of the HPD as the visit probability to each category [14]. HPD is not the same as the probability; it may become larger than 1 or less than −1 in value.

## Results

**Figure 6:**
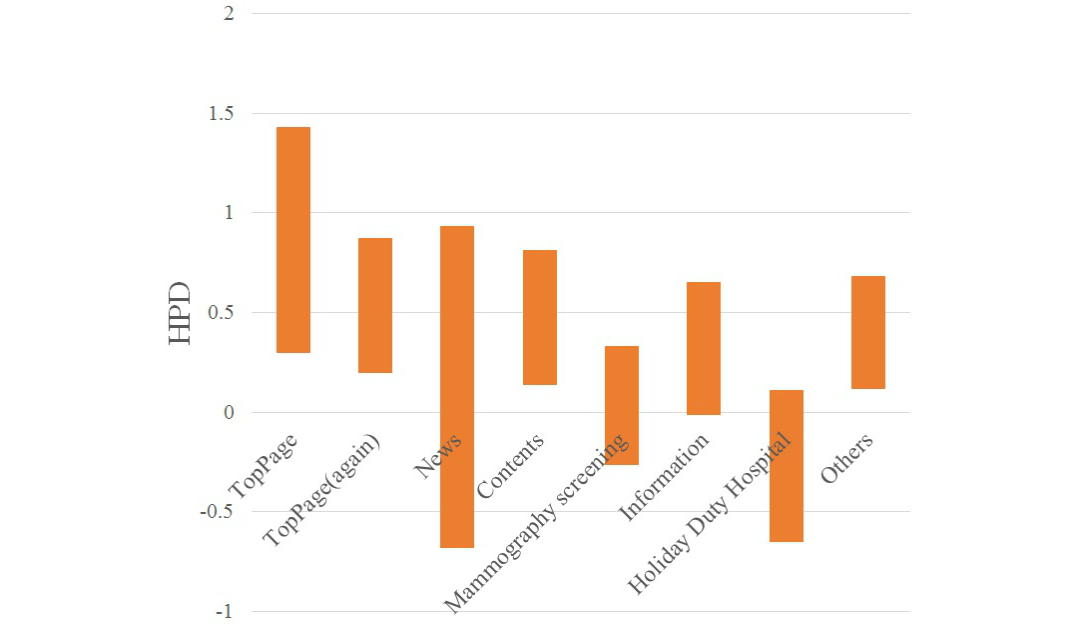
HPD of posterior distribution by keyword "clinic name".

**Figure 7:**
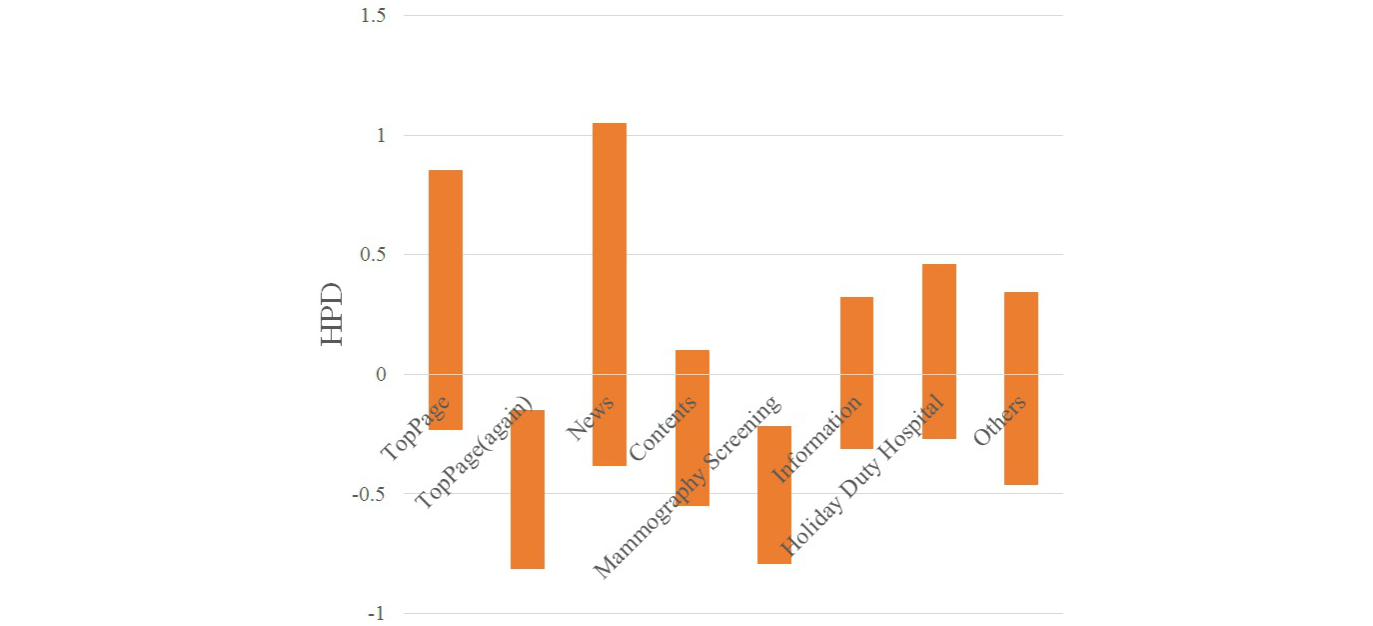
HPD of posterior distribution by keyword "clinic name + regional name".

**Figure 8:**
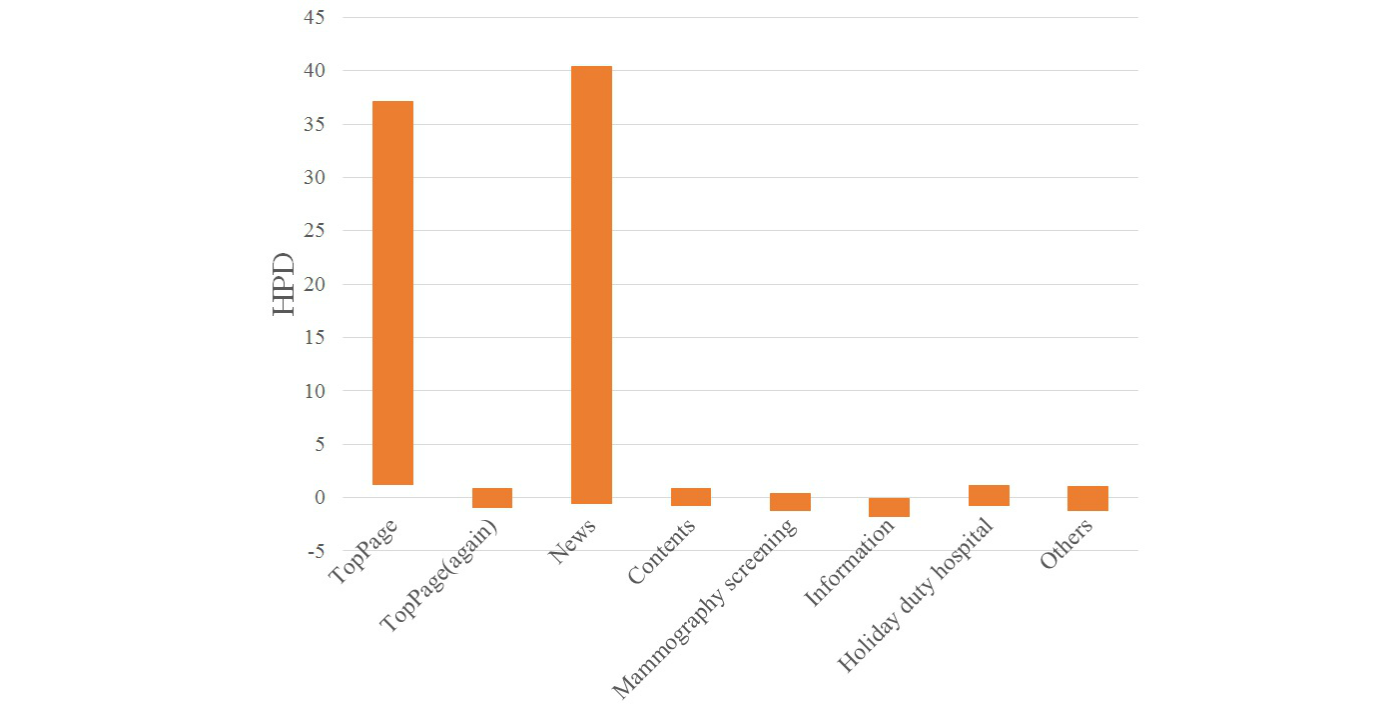
HPD of posterior distribution by keyword “clinic name + medical examination.”.

**Figure 9:**
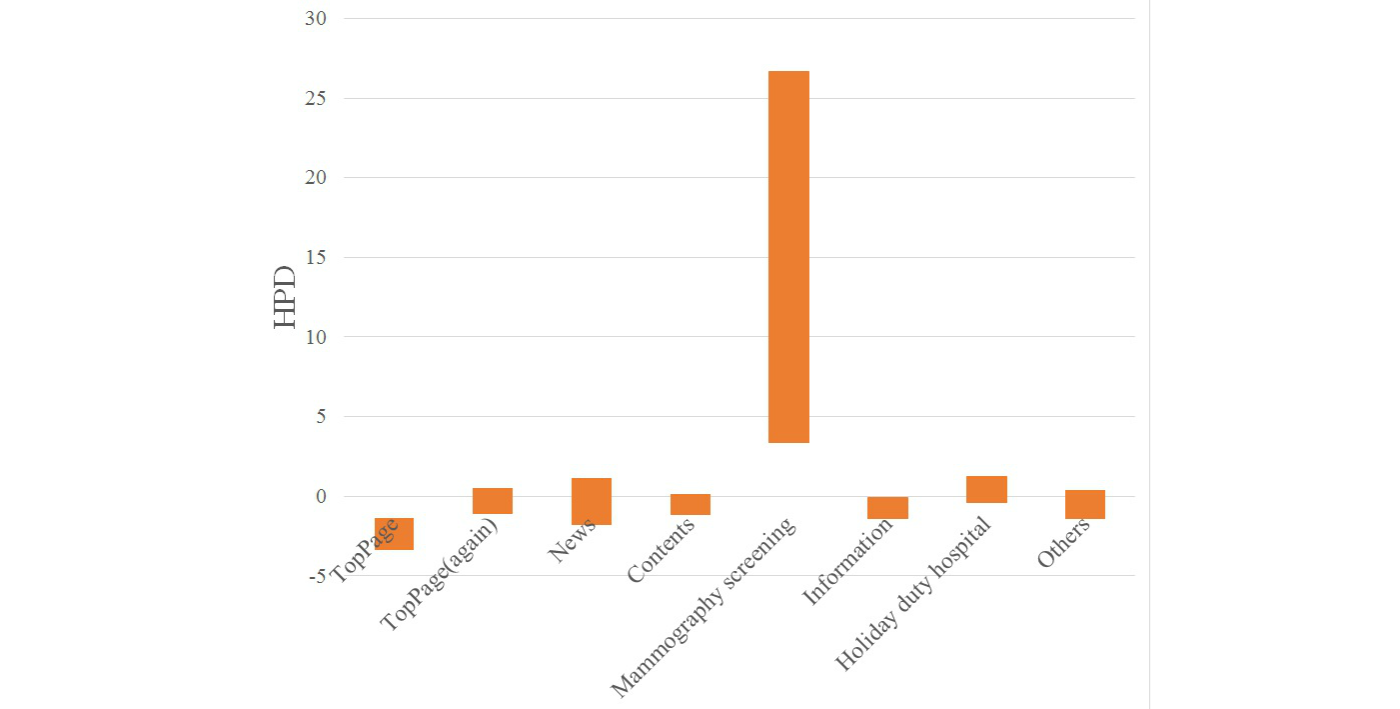
HPD of posterior distribution by keyword "mammography screening".

The statistical results for each keyword are shown in Tables 1-4 and Figures 6-9.

When a visitor referred to the keyword “clinic name,” the HPDs to the main page, the website (again), and the contents page were positive. When a visitor referred to the keyword “clinic name + regional name,” the HPDs to the website (again) and the mammography screening page were negative. When a visitor referred to the keyword “clinic name + medical examination,” the HPD to the main page was positive and that to the information page was negative. When a visitor referred to the keyword “mammography screening,” the HPD to the mammography screening was positive.

**Table 1:** Posterior distribution presumption result by keyword “clinic name”.

			95% HPD^a^ interval			
Contents	Posterior mean	SD^b^	2.50%	Median	97.50%	Convergence
Top page	0.85	0.29	0.3	0.85	1.43	○
Top page (again)	0.54	0.17	0.2	0.54	0.87	○
News	0.12	0.41	−0.68	0.11	0.93	○
Contents	0.48	0.17	0.14	0.47	0.81	○
Mammography screening	0.03	0.15	−0.26	0.04	0.33	○
Information	0.32	0.17	−0.01	0.32	0.65	○
Holiday duty hospital	−0.27	0.19	−0.65	−0.27	0.11	○
Others	0.27	0.2	−0.12	0.28	0.68	○

^a^HPD: highest posterior density.

^b^SD: standard deviation.

**Table 2:** Posterior distribution presumption result by keyword “clinic name + regional name”.

			95% HPD^a^ interval			
Contents	Posterior mean	SD^b^	2.50%	Median	97.50%	Convergence
Top page	0.31	0.28	−0.23	0.3	0.85	○
Top page (again)	−0.48	0.17	−0.81	−0.48	−0.15	○
News	0.33	0.37	−0.38	0.33	1.05	○
Contents	−0.23	0.16	−0.55	−0.23	0.1	○
Mammography screening	−0.5	0.15	−0.79	−0.5	−0.22	○
Information	0.01	0.16	−0.31	0.01	0.32	○
Holiday duty hospital	0.09	0.19	−0.27	0.09	0.46	○
Others	−0.06	0.2	−0.46	−0.06	0.34	○

^a^HPD: highest posterior density.

^b^SD: standard deviation.

**Table 3:** Posterior distribution presumption result by keyword “clinic name + medical examination”.

			95% HPD^a^ interval			
Contents	Posterior mean	SD^b^	2.50%	Median	97.50%	Convergence
Top page	17.01	9.61	1.26	15.96	37.08	○
Top page (again)	−0.02	0.45	−0.92	−0.01	0.86	○
News	13.94	12.05	−0.59	10.49	40.41	○
Contents	0.01	0.41	−0.78	0	0.85	○
Mammography screening	−0.41	0.4	−1.24	−0.39	0.34	○
Information	−0.88	0.43	−1.80	−0.86	−0.09	×
Holiday duty hospital	0.25	0.48	−0.71	0.25	1.17	○
Others	0.03	0.56	−1.17	0.06	1.06	○

^a^HPD: highest posterior density.

^b^SD: standard deviation.

**Table 4:** Posterior distribution presumption result by keyword “mammography screening”.

			95% HPD^a^ interval			
Contents	Posterior mean	SD^b^	2.50%	Median	97.50%	Convergence
Top page	−2.34	0.51	−3.35	−2.33	−1.37	○
Top page (again)	−0.27	0.4	−1.07	−0.27	0.51	○
News	−0.29	0.75	−1.79	−0.29	1.17	×
Contents	−0.5	0.33	−1.16	−0.5	0.14	○
Mammography screening	14.84	6.59	3.38	14.58	26.66	×
Information	−0.71	0.35	−1.41	−0.71	−0.05	×
Holiday duty hospital	0.41	0.4	−0.37	0.41	1.22	○
Others	−0.43	0.46	−1.38	−0.42	0.42	○

^a^HPD: highest posterior density.

^b^SD: standard deviation.

**Figure 10:**
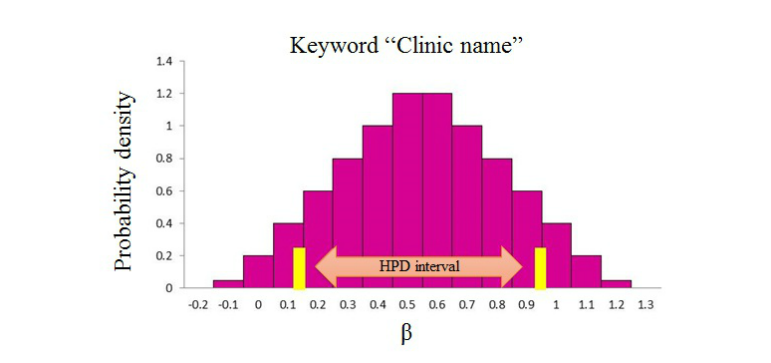
Posterior distribution by keyword "clinic name".

Next, we showed the results of the simulations regarding the time it took for a visitor to refer to the keyword “clinic name.” Figure 10 shows the presumed posterior distribution, and the horizontal axis is the value of parameter beta. The vertical axis is probability density. Posterior distribution obtained from this simulation was a unimodal distribution. The posterior distribution obtained by the vertical axis expressing probability density was a unimodal distribution.

**Figure 11:**
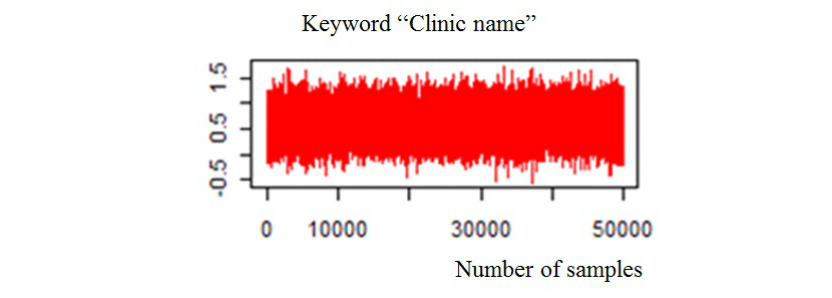
The simulation convergence situation by　keyword "clinic name".

Figure 11 shows the sampling convergence by MCMC, with the vertical axis as the beta value and the horizontal axis as the sampling number.

**Figure 12:**
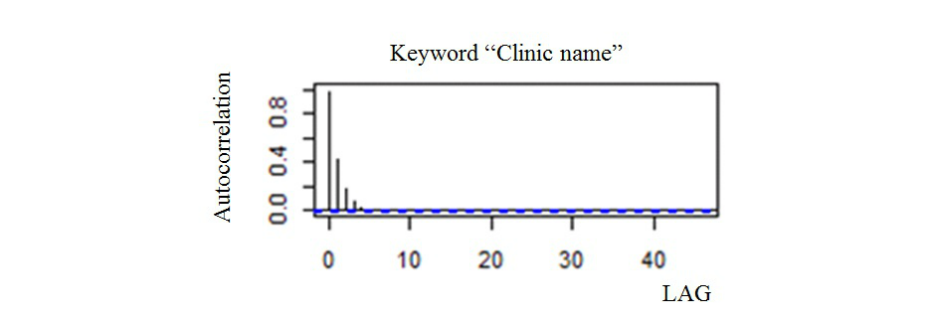
ACF by keyword "clinic name".

Figure 12 shows the ACF obtained by the simulation. For the keyword “clinic name,” the autocorrelation was small, and it was fully completed [15].

## Discussion

### Analysis of Searching Behavior of Medical Institution’s Website Visitors

From the MCMC results, the ACF converged on most pages. This means that we obtained consistent results. Therefore, we expect our results to be generally valid.

When a visitor referred to the keyword “clinic name,” the visit probability to the main, website (again), and contents pages addressing medical examinations was positive. Thus, search by keyword “clinic name” had the effect of increasing the probability of visits to the main, website (again), and contents pages. In particular, it is possible that the primary concern of a visitor who referred to a keyword “clinic name” was to reach the contents page addressing medical examinations. The visit probability to the holiday duty hospital page was negative. Visitors to the holiday duty hospital information page did not refer to a clinic name, and it is possible that many people visited this page from other linked pages.

When a visitor referred to the keyword “clinic name and regional name,” the visit probability to the website (again) and the mammography screening page was negative. Search by keyword “clinic name and regional name” had the effect of decreasing the probability of visits to the website (again) and mammography screening pages. The visit probability to the website (again) was also low. The visitor using this keyword did not visit the website for a second time within the same session, so, it is likely that they were uninterested in the mammography screening page.

When visitors referred to the keyword “clinic name and medical examination,” the visit probability to the main page was positive, and the visit probability to the information page was negative. Search by keyword “clinic name and medical examination” had the effect of increasing the probability of visits to the main page and decreasing the probability of the visits to the mammography screening page. Some visitors who visited the contents of the medical examination in the keywords had a low probability of visiting the contents page of the medical examination. In such instances, we thought the visitor did not get the information they wanted. We concluded that the website did not lead its visitors to the page having the information they required. We suggest that the hospital administration changes its webpage design to address this problem.

When visitors referred to the keyword “mammography screening,” the visit probability to the mammography screening page was positive. Thus, the website did lead visitors who visited by the keyword “mammography screening” to the page they wanted. Search by keyword “mammography screening” had the effect of increasing the probability of visits to the mammography page. This indicated that the visitors could arrive at the page that they wanted. In this area, medical institutions that have implemented mammography screening are not many. Therefore, the results are expected.

As the visit probability to the main and information pages was negative, we concluded that visitors had no interest in these pages. Information about access to the clinic was published on the website. As the visit probability to the website was low, we concluded that visitors who referred to the keyword “mammography screening” had not yet become patients of the clinic.

These results reveal that the tendency of the visit probabilities in each category was different for different keywords. Therefore, it is possible to increase the visit probability to the page a visitor wants by understanding the search behaviors based on visitor needs and therefore improve website effectiveness.

### Problems and Overview

This study identified 4 problems for consideration.

### A Setup of an Interest Level

Page view, although used, could not be reflected in the result beyond recording the presence or absence of visits to each page, not the presence or absence of browsing behavior. How much browsing by visitors actually reflects their needs is unclear. Therefore, a model analysis that would include inspection time by visitors would improve our ability to gauge visitor interest.

### Six-Month Study Period

The fact that medical institution patient numbers fluctuate with the seasons should be taken into account. In this research, the access log covered a period of 6 months only; therefore, the fluctuations in patient numbers by season were not considered. In the study of medical institution websites, seasonality has not been studied. As we believe that visitor access to website pages may also follow seasonal patterns, future research periods should also span one full year. Moreover, we think that a larger dataset is needed to accurately determine convergence in website access samples. Regardless of whether the amount of data used in this study was sufficient, we think that it is necessary to compare the analysis results using more data.

### Problem of a Prior Distribution Setup

In this study, because we had access only to data from the access log collection period, a noninformation prior distribution was assumed, so, no actual past data are reflected. To model a prior distribution, Ueda et al developed a prior distribution with high flexibility based on a nonparametric Bayesian model [15]. Bayesian estimation allows the determination of a posterior distribution, considering past data. We would like to consider using this technique for future research.

In this study, we used a noninformation prior distribution as the prior distribution. By setting the collected data with the prior distribution, we will be able to build a new model of medical institution webpage browsing behavior. In addition, using beta values obtained from improved prior distributions to estimate the behavior of website visitors, it becomes possible to build a website appropriate to medical institutions.

### Problem of Model Selection

Although the binomial probit model was used in this research, we could not verify the validity of the model. As the logit model, the classic Bayesian model, the nonparametric Bayesian model, and so forth are proposed in the literature as discrete selection models for use with a Bayesian approach, it is necessary to validate our model by comparing it with those of others [15-17]. One feature of medical institutions is that patient region and age groups differ by hospital scale, department, and region. In our research, because the only clinic website we targeted was the one near Sapporo, we could not address the characteristics of regionality or hospital scale. To better identify the browsing characteristics of visitors to the websites of many medical institutions, we would like to analyze other departments, regional areas, hospital scale, and so forth.

　Recently, branding has become a marketing technique for hospital networks, and many patients select hospitals by recognizing their brands. In these situations, some hospitals are adopting a differentiated marketing strategy. Moreover, they are beginning to undertake customer relationship management (CRM), recognizing the lifetime value of a customer. As a hospital is an organization providing medical treatment as a service, it essentially has the same marketing challenges of any other company, while varying to a considerable degree in terms of the services provided for the public benefit. However, research by Kim states that the health care field can effectively apply CRM as well as any other field. The Bayesian approach used in this research is also a useful technique in CRM. As it is possible to perform heterogeneity modeling between consumers, this tool can be developed as one of the database marketing strategies for medical treatment [18].

　For the modeling of heterogeneity among consumers, purchasing history data analysis, estimating heterogeneous price thresholds, and e-commerce site visitor behavior analysis has been conducted recently. However, behavior analysis on the websites of medical institutions remains unstudied [19-21].

　Selection of medical institutions, as in the case of selecting products and services other than medical services, is affected by such competitive relationships between the patient’s preferences and brand. In medical institution marketing activities, it is very important to know the variables. Therefore, it is necessary to further ascertain the heterogeneity between patients.

### Conclusion

To clarify the information that citizens want when searching the Web, we developed a searching behavior model for visitors to a medical institution's website using a Bayesian approach and determined the visit probability to each category of interest, classified by search keyword. We targeted the website access log of a clinic near Sapporo, for the January 1 to June 31, 2011 period and determined the visit probability to each category using the predetermined search keywords. In the case of the keyword “clinic name,” the visit probability to the website, the website (again), and the contents page for medical examination was positive. For the holiday person-on-duty page, visit probability was negative. In the case of the keyword “clinic name and regional name,” the visit probability to the website (again) and the mammography screening page was negative. In the case of the keyword “clinic name + medical examination,” the visit probability to the website was positive, and the visit probability to the information page was negative. When visitors referred to the keywords “mammography screening,” the visit probability to the mammography screening page was positive. Further analysis for not only the clinic website but also various other medical institution websites is necessary to build a general inspection model for medical institution websites; we want to consider this in future research. In addition, we hope to use the results obtained in this study as a prior distribution for future work and to conduct higher precision analysis.

## Appendix

Multimedia Appendix 1 R-code.

## Abbreviations

ACF: autocorrelation function
CRM: customer relationship management
MCMC: Markov Chain Monte Carlo
HPD: highest posterior density
