# Supplementary material for: Behavioral Analysis of Visitors to a Medical Institution’s Website Using Markov Chain Monte Carlo Methods
Source: J Med Internet Res. 2016 Jul 25;18(7):e199. doi: 10.2196/jmir.5139 (PMC4977421; doi:10.2196/jmir.5139)
Supplement: Multimedia Appendix 1 [file jmir_v18i7e199_app1.pdf]

```
# read the data
Clinicname <- read.table("Clinicname.csv",sep=",",header=T)
summary(Clinicname)

# MCMCpack
library(MCMCpack)
pro.post1                                                                <-
MCMCprobit(Clinicname~toppage+toppageagain+news+contents+mammogra
phy+infomation+Holiday+others, data=Clinicname,
burnin = 5000, mcmc = 50000, b0 = 0, B0 = 0.001)
summary(pro.post1)

# figure 4.1
plot(pro.post1)
# bayesm
library(bayesm)
Data2<-list(X=cbind(Clinicname$toppage,
Clinicname$Toppageagain,Clinicname$news,Clinicname$contents,Clinicname$
mammography,Clinicname$infomation,Clinicname$Holiday,Clinicname$others),
y=Clinicname$Clinicname)
mcmc2 <- list(R=10000,keep=1)
pro.post2 <- rbprobitGibbs(Data=Data2,Mcmc=mcmc2)
summary(pro.post2$betadraw)
# figure 4.2
plot(pro.post2$betadraw)
```
